# Promoting positive masculinities among young people in Stockholm, Sweden. A mixed-methods study

**DOI:** 10.1093/eurpub/ckac131.023

**Published:** 2022-10-25

**Authors:** M Salazar, A Cerdán-Torregrosa

**Affiliations:** 1 Global and Sexual Health Research Group, Department of Global Public Health, Karolinska Institutet, Stockholm, Sweden; 2 Department of Community Nursing, Preventive Medicine and Public Health and History of Science, University of Alicante, Alicante, Spain

## Abstract

**Background:**

Despite policies aiming to curtail men’s violence against women (VAW) in Sweden, one in three women have experience physical/sexual VAW. Promoting anti-VAW masculinities among young men is a key intervention to reduce VAW; yet little is known about what actions could be used to effectively do so in Sweden. This study aims to: 1. Identify actions that young people (men and women), and stakeholders believe can be used to promote anti-VAW masculinities and 2. Quantify the relationship, coherence and patterns of importance and applicability between the different identified actions.

**Methods:**

A mixed-methods study was conducted in Stockholm in 2019. In-depth interviews with young people aged 18-24 years (men= 16, women=12) and stakeholders (n = 12) were used to identify actions to promote anti-VAW masculinities. Then, an online survey with 83 people (77 young people) was conducted asking participants to sort the actions and rate them in terms of importance and applicability. Multidimensional scaling and hierarchical cluster analysis were used to create clusters maps. Each cluster was rated in terms of importance and applicability.

**Results:**

Six clusters were identified: 1.own self-reflection and change, 2. actions in leisure-cultural spaces, 3. mandatory education on gender-VAW, 4. positive role models in public arenas, 5. support civil society and 6. strengthen government, police, and legal response. The clusters of mandatory education on gender-VAW and own self-reflection and change were rated higher in importance (mean 5.1 and 4.8 respectively). Mandatory education on gender-VAW and actions in leisure-cultural spaces were rated higher in applicability (mean 4.6 and 4.7 respectively). Correlation between importance and applicability was low (rho=0.16).

**Conclusions:**

Promoting anti-VAW masculinities to tackle VAW should be done in multiple arenas. Mandatory education on gender-VAW in schools and leisure spaces are key strategies to promote anti-VAW masculinities.

**Key messages:**

• Preventing VAW by focusing on masculinities requires the involvement of various social spheres.

• Mandatory education on gender and VAW is considered key in curtailing men’s VAW in Sweden.

